# Delayed vasocutaneous fistula following vasectomy associated with retained silk ligature: a case report with clinicopathological correlation

**DOI:** 10.1186/s12894-026-02313-z

**Published:** 2026-08-14

**Authors:** Senthil Kumar Thiagarajan, Dayakar Surati, Ravi Chandu Ponugoti, Balaji Subramaniam

**Affiliations:** Department of Urology, SRM Medical College Hospital and Research Centre, SRM Institute of Science and Technology, Chennai, Tamil Nadu 603211 India

**Keywords:** Vasectomy, Vasocutaneous fistula, Chronic inflammation, Retained suture material, Case report

## Abstract

**Background:**

Vasectomy is a safe and highly effective method of permanent male contraception. Delayed vasocutaneous fistula is an exceptionally uncommon complication, and its diagnosis requires careful correlation of clinical, laboratory, operative and pathological findings. We report a delayed vasocutaneous fistula associated with retained silk ligature at the previous vasectomy site and discuss the diagnostic approach and probable mechanism.

**Case presentation:**

A 43-year-old man presented with a two-month history of persistent whitish discharge from the root of the left hemiscrotum, three years after bilateral no-scalpel vasectomy. Clinical examination demonstrated a sinus opening with an underlying thickened vas deferens, Microscopic examination of the discharge revealed occasional spermatozoa. Preoperative ultrasonography demonstrated a hypoechoic tract extending towards the previous vasectomy site with adjacent inflammatory changes. During surgical exploration, a tract terminating at the previously silk-ligated proximal vasal stump was identified. The tract together with the involved vasal segment was excised en bloc. Histopathological examination demonstrated a squamous epithelium-lined sinus tract containing retained suture material, surrounded by chronic inflammatory infiltrates, composed predominantly of lymphocytes and plasma cells together with an adjacent fragment of vas deferens. These findings supported chronic inflammation associated with retained suture material. The patient remained asymptomatic without recurrence during follow-up.

**Conclusion:**

This case demonstrates that the diagnosis of delayed vasocutaneous fistula should be established through correlation of clinical findings, microscopic identification of spermatozoa, operative findings and histopathological examination rather than histopathology alone. Retained non-absorbable suture material, consistent with the previously documented silk ligature, may contribute to persistent chronic inflammation and delayed fistula formation following vasectomy.

## Background

Vasectomy is one of the most effective methods of permanent male contraception, with an estimated contraceptive efficacy exceeding 99% and millions of procedures performed worldwide each year. Owing to its simplicity, safety profile and cost-effectiveness, it remains a widely accepted method of sterilisation [1, 2]. Most complications are minor and include haematoma, wound infection, sperm granuloma and chronic post-vasectomy pain syndrome. Delayed fistulous complications involving the vas deferens are exceptionally uncommon [3–7].

Vasocutaneous fistula represents an abnormal epithelialised communication between the vas deferens and the overlying skin. The published literature is limited to isolated case reports and a recent review summarising rare post-vasectomy fistulous complications. Because published evidence is limited to isolated case reports, the diagnostic approach, underlying pathogenesis and optimal management remain incompletely characterised [8, 9].

We report a delayed vasocutaneous fistula occurring three years after bilateral no-scalpel vasectomy. Compared with previously reported cases, this case combines clinical findings, laboratory analysis, operative observations and histopathological examination to support the diagnosis and demonstrates retained black braided suture, consistent with the previously documented silk ligature, as the plausible substrate for persistent chronic inflammation contributing to delayed fistula formation.

## Case presentation

A 43-year-old man presented to our outpatient urology clinic with a two-month history of persistent whitish discharge from the root of the left hemiscrotum. The discharge was spontaneous, non-foul smelling, and occasionally increased with local pressure. He denied fever, urinary symptoms, haematuria, haematospermia, scrotal trauma, or previous episodes of similar discharge.

Three years earlier, he had undergone bilateral no-scalpel vasectomy for permanent contraception at another institution. According to the available operative details, the procedure was performed through bilateral scrotal puncture sites under local anaesthesia. The proximal vasal stump on each side was ligated using non-absorbable silk sutures, monopolar electrocautery was applied, and both vasal ends were ligated according to the institutional technique. The postoperative recovery was uneventful. Post-vasectomy semen analysis had not been performed.

**Fig. 1:**
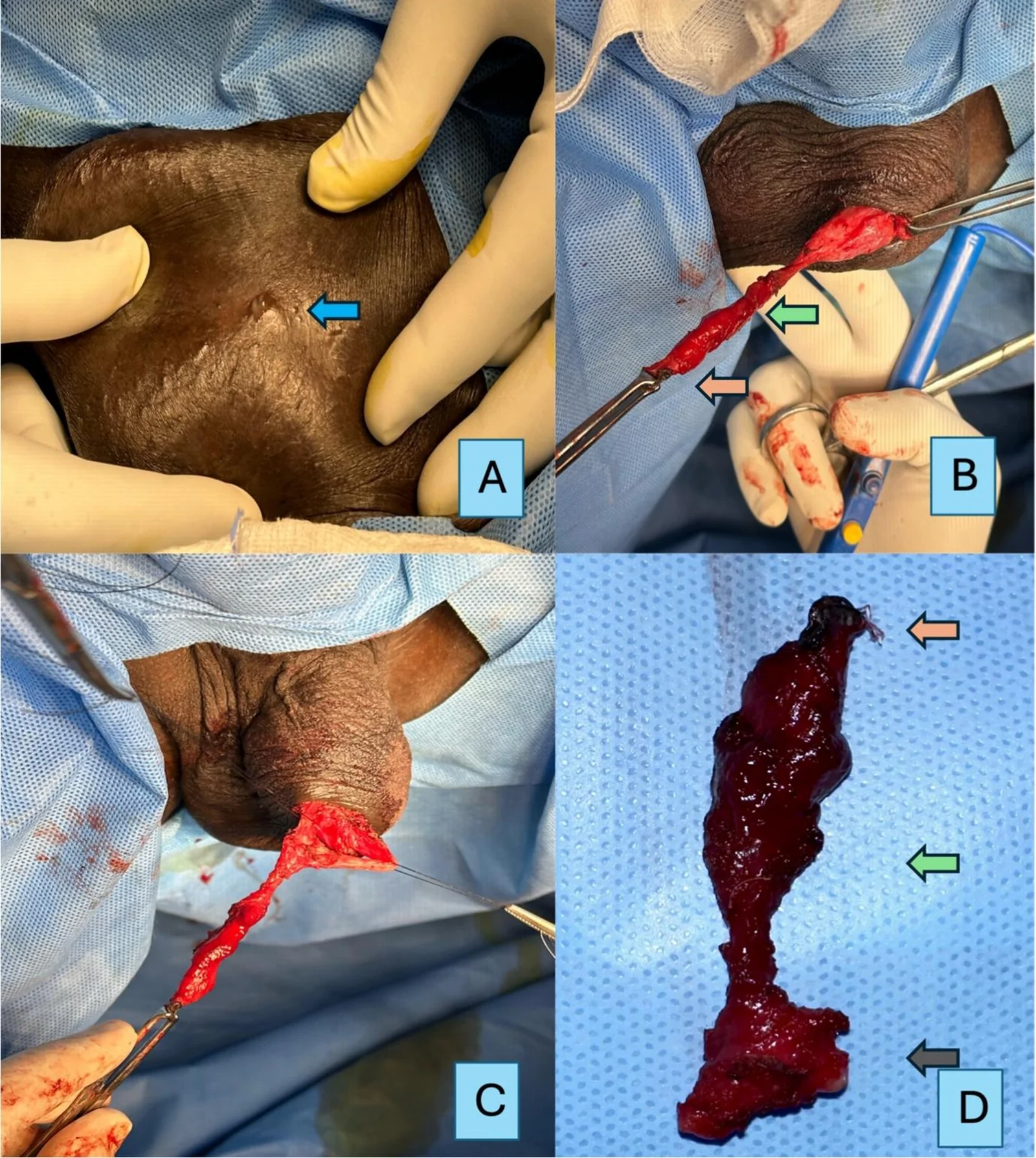
Clinical and operative findings. Legend : **A** Preoperative clinical photograph demonstrating a small external sinus opening at the root of the left hemiscrotum. **B** Intraoperative dissection demonstrating the epithelialized fistulous tract extending towards the previous vasectomy site. **C** En bloc excision of the fistulous tract together with the involved vasal segment. **D** Gross specimen demonstrating the en bloc excised epithelialized fistulous tract with the attached involved vasal segment

Examination revealed a 2-mm sinus opening at the root of the left hemiscrotum with expression of whitish discharge on gentle compression. (Fig. 1A) A thickened left vas deferens was palpable immediately deep to the sinus. There was no surrounding cellulitis, fluctuance, scrotal edema, or inguinal lymphadenopathy. The rest of the scrotal examination was unremarkable.

Microscopic examination of the expressed discharge demonstrated occasional spermatozoa together with inflammatory cells. Routine haematological investigations, serum biochemical parameters and urinalysis were within normal limits.

Preoperative high-resolution ultrasonography demonstrated a hypoechoic tract extending from the cutaneous opening towards the previous left vasectomy site. The adjacent proximal spermatic cord structures appeared thickened, consistent with chronic inflammatory change. Although the original ultrasonographic images and report were unavailable for inclusion, these findings supported the preoperative suspicion of a fistulous communication.

Taken together, the persistent sinus, identification of spermatozoa within the discharge, and ultrasonographic findings raised a strong clinical suspicion of a vasocutaneous fistula involving the previously ligated vas deferens.

Surgical exploration confirmed a continuous epithelialised tract extending from the cutaneous opening to the previously ligated proximal vasal stump. A retained black braided suture, consistent with the previously documented non-absorbable silk ligature, was embedded within dense fibrotic tissue at the point of communication. The tract terminated at this site without evidence of abscess formation or extension beyond the vasectomy stump. The involved vasal segment and fistulous tract were excised en bloc. The remaining healthy proximal vas was re-ligated using absorbable suture (Polyglactin) before wound closure. (Fig. 1B-D)

**Fig. 2:**
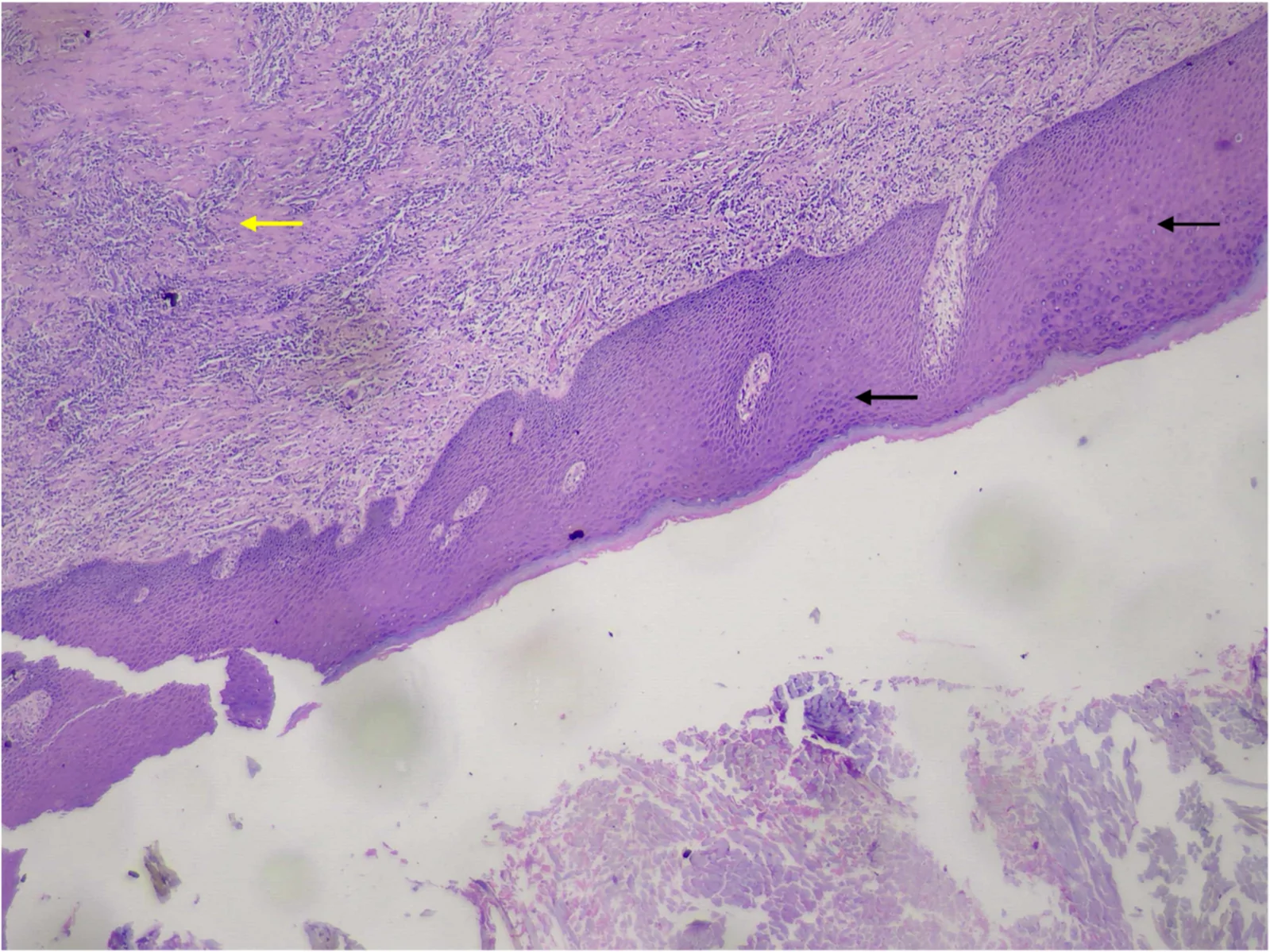
Histopathological examination of the excised specimen (H&E, x100). Legend : Photomicrograph demonstrating a squamous epithelium-lined sinus tract (black arrows) within fibrocollagenous tissue. The surrounding stroma shows chronic inflammatory infiltrates composed predominantly of lymphocytes and plasma cells (yellow arrow). Histopathological examination of the complete specimen demonstrated a chronic epithelialized tract containing retained suture material with surrounding chronic inflammatory infiltrates and an adjacent fragment of vas deferens, as described in the main text

The excised specimen was fixed in 10% neutral buffered formalin, routinely processed, paraffin embedded, sectioned, and stained with haematoxylin and eosin. Histopathological examination demonstrated a squamous epithelium-lined sinus tract containing retained suture material with surrounding chronic inflammatory infiltrates composed predominantly of lymphocytes and plasma cells, together with an adjacent fragment of vas deferens lined by pseudostratified columnar epithelium (Fig. 2). These findings supported the operative diagnosis by confirming an epithelialized tract with retained suture material and chronic inflammation adjacent to the vas deferens.

The diagnosis of vasocutaneous fistula was established intraoperatively by direct visualization of anatomical continuity between the epithelialized cutaneous tract and the previously ligated proximal vasal stump. Histopathological examination supported the operative findings by confirming the excised epithelialized tract and adjacent vas deferens but did not independently establish the fistulous communication.

The postoperative course was uneventful. At one week, the wound had healed satisfactorily. At one month and three months of follow-up, the patient remained asymptomatic with complete resolution of the discharge and no clinical evidence of recurrence.

## Discussion

Delayed vasocutaneous fistula is an exceptionally uncommon late complication of vasectomy, with the published literature largely confined to isolated case reports and a recent narrative review. Compared with previous reports, the present report not only documents delayed vasocutaneous fistula but also illustrates a structured diagnostic approach based on integration of clinical examination, microscopic identification of spermatozoa within the sinus discharge, supportive ultrasonographic findings, operative demonstration of fistulous continuity, and histopathological examination showing retained suture material associated with chronic inflammatory infiltrates. Although these findings are not specific for any particular inflammatory mechanism, when interpreted together with the operative identification of retained black braided suture at the previous vasectomy site and the documented use of non-absorbable silk ligatures during the index vasectomy, they support the hypothesis that chronic suture-associated inflammation contributed to progressive epithelialization of the tract and delayed fistula formation [8, 9]. 

Establishing the diagnosis of vasocutaneous fistula can be challenging because no single investigation is independently diagnostic. Histopathological examination may demonstrate a sinus tract, chronic inflammation, retained suture material, and adjacent vas deferens; however, these findings alone do not conclusively establish a fistulous communication. Importantly, in the present case, histopathology served to corroborate the intraoperative findings by confirming the epithelialized tract, retained suture material, and adjacent vas deferens, whereas the anatomical continuity of the fistula was established during surgical exploration. Similarly, the identification of spermatozoa within the sinus discharge strongly suggests communication with the vas deferens but does not localize the anatomical pathway. In the present case, the diagnosis was established through concordant evidence comprising persistent sperm-containing sinus discharge, supportive ultrasonographic findings, intraoperative demonstration of a continuous tract extending to the previously ligated proximal vasal stump, and histopathological findings demonstrating an epithelialized tract containing retained suture material with chronic inflammatory infiltrates adjacent to the vas deferens. This integrated clinicopathological approach provides a more robust diagnostic framework than reliance on any individual modality alone [8, 9]. To our knowledge, previous reports have not described diagnosis based on systematic integration of clinical, laboratory, operative and histopathological findings in the absence of a single definitive diagnostic test.

**Fig. 3:**
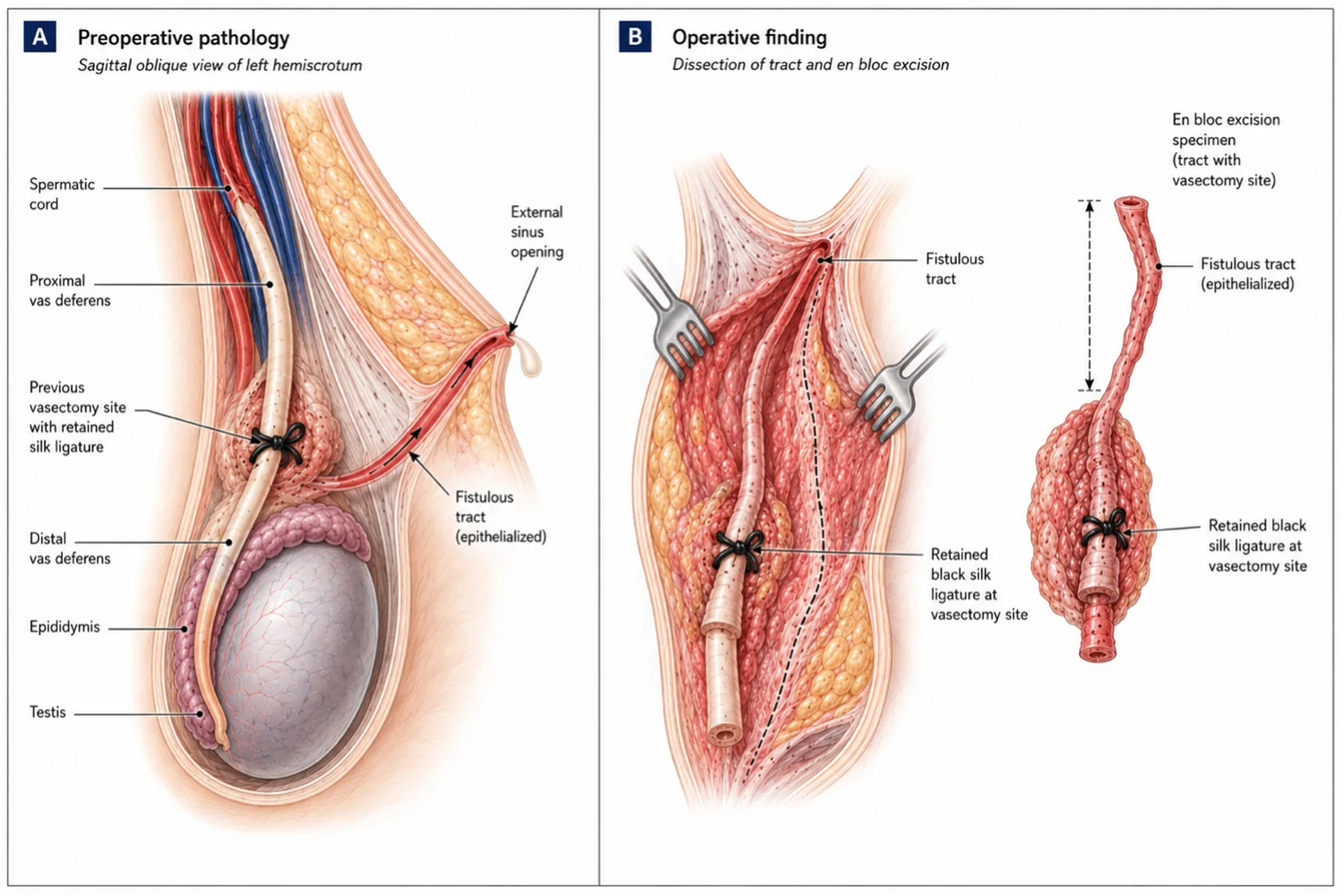
Proposed mechanism of delayed vasocutaneous fistula associated with retained non-absorbable suture material (schematic illustration of proposed mechanism). Legend : **A** Schematic illustration demonstrating a retained non-absorbable silk ligature at the previous vasectomy site with an epithelialized fistulous tract extending to the external sinus opening at the root of the left hemiscrotum. **B** Operative schematic demonstrating dissection of the fistulous tract, complete en bloc excision with the involved vasal segment, and the retained black braided suture consistent with previously documented non-absorbable silk ligature at the vasectomy site

The precise pathogenesis of delayed vasocutaneous fistula remains uncertain and is likely multifactorial. Proposed mechanisms include persistent sperm leakage with granuloma formation, chronic low-grade infection, spontaneous recanalization, foreign body reaction, and impaired wound healing following vasectomy. In the present case, several operative and histopathological findings support the hypothesis of chronic suture-associated inflammation contributing to delayed fistula formation. Surgical exploration demonstrated a retained non-absorbable silk ligature, consistent with the previously documented non-absorbable silk ligature, embedded within dense fibrotic tissue at the point where the fistulous tract communicated with the proximal vasal stump. Histopathological examination further demonstrated retained suture material associated with chronic inflammatory infiltrates within a mature epithelialised tract. Although a direct causal relationship cannot be established from a single case, these concordant findings suggest that persistent chronic inflammation associated with retained suture material may have contributed to progressive epithelialisation of the tract and delayed fistula formation over the three-year interval following vasectomy [3–6, 8]. The proposed mechanism underlying delayed fistula formation is illustrated schematically in Fig. 3.

The observations in our patient can be better appreciated when compared with the limited published reports of post-vasectomy vasocutaneous fistula. Only a limited number of vasocutaneous fistulas following vasectomy have been reported in the literature. Gaden and Kasraie described a patient presenting nine years after vasectomy with intermittent scrotal discharge containing spermatozoa, thereby establishing sperm microscopy as an important diagnostic clue, as shown in Table 1. However, the underlying anatomical basis for the fistulous communication was not identified, and operative or histopathological correlation was not described. More recently, Lamoury et al. systematically reviewed the spectrum of vasocutaneous fistulas and highlighted the rarity and heterogeneity of these lesions, while emphasising that their pathogenesis remains poorly understood. In contrast, the present case combines clinical examination, sperm microscopy, supportive ultrasonography, operative demonstration of tract continuity, and histopathological findings, thereby providing a comprehensive clinicopathological correlation. Furthermore, the identification of retained black braided suture, consistent with the previously documented silk ligature at the site of communication offers a plausible anatomical substrate for delayed fistula formation that has not been clearly documented in previous reports [8, 9]. Other rare fistulous complications of vasectomy, including vasovenous fistula, have also been described, illustrating the diverse spectrum of delayed aberrant communications that may occur following vasal surgery. However, unlike the internal vasovenous communication reported by Rajan et al., the present case represents a true epithelialised communication between the vas deferens and the overlying skin [10].

**Table 1:** Published reports of vasocutaneous fistula following vasectomy and comparison with the present case

Author	Interval	Presentation	Key diagnostic evidence	Proposed mechanism	Treatment	Outcome
Gaden et al. [9]	9 years	Scrotal discharge	Sperm in discharge	Recanalization suspected	Excision planned	NR
Present case	3 years	Persistent sinus discharge	Sperm microscopy + USG + operative continuity + histopathology	Retained suture material (consistent with silk ligature) with chronic inflammation	Complete excision	No recurrence

From a clinical perspective, persistent or recurrent scrotal discharge following vasectomy should not be attributed solely to superficial wound infection or chronic sinus formation. Although exceedingly uncommon, vasocutaneous fistula should be considered in the differential diagnosis, particularly when the discharge is persistent or contains spermatozoa on microscopic examination. While ultrasonography may support the diagnosis by demonstrating a tract or associated inflammatory changes, definitive diagnosis often requires surgical exploration to establish anatomical continuity with the vas deferens. Complete excision of the epithelialised tract together with the involved vasal segment and any retained foreign material appears to provide definitive treatment and was associated with complete resolution without recurrence in our patient. Adherence to meticulous vasectomy technique and minimization of permanent foreign material, where appropriate, may further reduce the risk of delayed inflammatory complications [3, 8, 11, 12]. 

This report has several limitations inherent to a single-case study. First, the ultrasound examination performed during the original clinical evaluation contributed to preoperative assessment; however, the original images were unavailable for independent review. Second, microscopy performed during the original evaluation demonstrated occasional spermatozoa, although a contemporaneous photomicrograph was unavailable. Finally, although the retained suture material and associated chronic inflammatory findings strongly suggest a chronic inflammation-mediated mechanism, a definitive causal relationship cannot be established from a single observation. Nevertheless, the diagnosis in the present case was supported by concordant clinical, laboratory, operative, and histopathological findings, and the patient’s complete resolution following excision further reinforces the proposed diagnosis and management strategy.

## Conclusion

Delayed vasocutaneous fistula is an exceptionally rare late complication of vasectomy that requires a high index of suspicion in patients presenting with persistent scrotal sinus discharge. This case demonstrates that diagnosis is best established through integration of clinical findings, sperm microscopy, supportive imaging, operative confirmation, and histopathological examination rather than reliance on any single modality. The intraoperative identification of retained black braided suture, consistent with the previously documented non-absorbable silk ligature, together with histopathological evidence of retained suture material associated with chronic inflammatory infiltrates, supports the hypothesis that chronic suture-associated inflammation contributed to delayed fistula formation. However, causality cannot be definitively established from a single case. Complete surgical excision of the fistulous tract, involved vasal segment, and retained foreign material resulted in durable resolution. This report adds to the limited literature by providing comprehensive clinicopathological correlation and proposing a diagnostic framework for this uncommon complication.

## Data Availability

The data supporting the findings of this case report are included within this published article. Additional de-identified clinical information is available from the corresponding author upon reasonable request, subject to institutional policies and patient confidentiality requirements.

## Abbreviations

H&E: Hematoxylin and Eosin
